# Alterations in the functional neural circuitry supporting flexible choice behavior in autism spectrum disorders

**DOI:** 10.1038/tp.2016.161

**Published:** 2016-10-11

**Authors:** A-M D'Cruz, M W Mosconi, M E Ragozzino, E H Cook, J A Sweeney

**Affiliations:** 1 Institute for Juvenile Research, University of Illinois at Chicago, Chicago, IL, USA; 2 Department of Psychology, University of Illinois at Chicago, Chicago, IL, USA; 3 Shiefelbusch Institute for Life Span Studies and Clinical Child Psychology Program, University of Kansas, Lawrence, KS, USA; 4 Department of Psychiatry, University of Cincinnati, Cincinnati, OH, USA

## Abstract

Restricted and repetitive behaviors, and a pronounced preference for behavioral and environmental consistency, are distinctive characteristics of autism spectrum disorder (ASD). Alterations in frontostriatal circuitry that supports flexible behavior might underlie this behavioral impairment. In an functional magnetic resonance imaging study of 17 individuals with ASD, and 23 age-, gender- and IQ-matched typically developing control participants, reversal learning tasks were used to assess behavioral flexibility as participants switched from one learned response choice to a different response choice when task contingencies changed. When choice outcome after reversal was uncertain, the ASD group demonstrated reduced activation in both frontal cortex and ventral striatum, in the absence of task performance differences. When the outcomes of novel responses were certain, there was no difference in brain activation between groups. Reduced activation in frontal cortex and ventral striatum suggest problems in decision-making and response planning, and in processing reinforcement cues, respectively. These processes, and their integration, are essential for flexible behavior. Alterations in these systems may therefore contribute to a rigid adherence to preferred behavioral patterns in individuals with an ASD. These findings provide an additional impetus for the use of reversal learning paradigms as a translational model for treatment development targeting the domain of restricted and repetitive behaviors in ASD.

## Introduction

Much research has focused on social deficits in autism spectrum disorders (ASD), but understanding of the restricted and repetitive behaviors symptom domain remains limited, despite the significant burden it places on affected individuals and their caregivers.^1, 2^ A neurocognitive deficit in disengaging from preferred behavioral patterns may contribute to this behavioral aspect of ASD.^3, 4, 5^ Because few treatment options for behavioral rigidity in ASD are currently available, defining the neural substrate of behavioral inflexibility has the potential to inform new treatment targets for this understudied feature of the disorder.

Reversal learning tasks provide a well-established and translational approach to examining flexible choice behavior. In contrast to extradimensional set-shifting tasks such as the Wisconsin Card Sorting Test^6^ in which the criterion for choosing a correct response might switch from color, to shape, to location, reversal learning tasks assess simple intradimensional shifts in behavior, for example, shifting from choosing one spatial location to another. This is accomplished by requiring subjects to learn a behavioral response using performance feedback, and then to reverse that response to an alternative option when a learned response preference is no longer the correct choice. Importantly, studies of reversal learning are readily conducted in rodent models, and thus are a useful methodology for translational approaches assessing potential mechanistic neurobiological models of behavioral inflexibility and evaluating drug effects on behavioral deficits (Brown, Amodeo, Sweeney, and Ragozzino, 2012; Ghahremani *et al.*;^7^ Glascher *et al.*;^8^ M. E. Ragozzino, Mohler, Prior, Palencia, and Rozman, 2009). The two-choice and multi-choice reversal learning studies presented here were designed to have strong parallels with T-maze and radial maze studies of reversal learning in rodents.

Few behavioral studies have examined reversal learning in ASD. Most have used small samples of young children who showed alterations in the ability to learn an initial response pattern, which complicates assessment of the ability to reverse preferences from that response.^9, 10^ In two recent studies of older individuals with an ASD, we demonstrated significant reversal learning and set shifting deficits that in both cases were related to clinical ratings of rigid and repetitive behavior.^3, 11^

To date, there has only been one neuroimaging study of reversal learning in ASD.^12^ In this study, male adolescents performed a two-choice probabilistic reversal learning task. In probabilistic reversal learning, reinforcement cues are not always accurate because misleading negative feedback is provided for some proportion of correct response trials. During probabilistic reversal learning, ASD individuals exhibited reduced activation of the medial prefrontal cortex and precuneus, consistent with past studies demonstrating these brain regions may be part of a larger neural system that is critical for processing negative feedback and facilitating a shift to an optimal response.^7, 13^ Although the use of a probabilistic reinforcement schedule can be informative, it confounds whether deficits exist in ASD when shifting behavior in response to accurate or inaccurate reinforcement cues. Crucially, there have been no neuroimaging studies of non-probabilistic reversal learning in ASD to date, and as such, the brain circuits responsible for disruptions in simple flexible choice behavior have not been identified.

A number of brain regions are known to support flexible behavioral control, and thus are potential areas where dysfunction could lead to reduced behavioral flexibility in ASD. Dorsal frontal systems, including cognitive and motor subdivisions of anterior cingulate, premotor and dorsolateral prefrontal cortex, and dorsal regions of posterior parietal cortex control the inhibition of prepotent response tendencies, and the subsequent planning and initiation of new, contextually dependent behaviors.^14, 15, 16, 17, 18^

Flexible behavior requires not only the ability to make decisions to change learned response patterns, but also to recognize changes in response contingencies that cue a need for such alterations in behavior. Unexpected nonreinforcement for a learned response elicits a negative reward prediction error signal that propagates from the midbrain to the nucleus accumbens, and serves an important role in facilitating adaptive changes in behavior based on performance feedback.^19^ Human neuroimaging studies have shown increased activation in ventral striatum and ventromedial frontal cortex in response to unexpected negative feedback.^8, 20, 21^ Functional magnetic resonance imaging (fMRI) studies of reversal learning thus allow for the evaluation of brain systems supporting decision-making and response planning as well as those that respond to unexpected non-reinforcement for learned response preferences.

Reversal learning paradigms cannot only assess flexible choice behavior, but also the impact of uncertainty of the outcomes of future choices on behavioral flexibility. In a two-choice reversal learning task, participants are presented with two response options.^22^ Once one response is no longer correct, the alternative response is certain to be the correct choice. When the number of response options is increased, participants can no longer be certain of the new correct choice, and a decision from available choice options is required. Using a four-choice reversal learning task during fMRI with typically developing individuals, we have shown that activation is increased in both dorsal and ventromedial frontal systems when the correct choice after reversal is uncertain.^20^

In the current study, individuals with ASD and matched typically developing control participants performed two- and four-choice reversal learning tasks during fMRI. We performed analyses designed to test two potential neurobehavioral mechanisms of behavioral rigidity in ASD: (1) impairment in premotor, prefrontal and parietal cortices, and in dorsal striatum, regions that are important in implementing cognitive changes in behavioral set (response choice and planning mechanisms); and (2) impairment in ventral striatum and the affective division of anterior cingulate cortex, which are associated with recognizing and responding to changes in reinforcement contingencies that motivate individuals to change behavior (reinforcement learning mechanisms).

## Materials and methods

### Study participants

Seventeen individuals with an ASD (5 females) and 23 typically developing controls (5 females) participated in the study (Table 1). Individuals with an ASD were recruited from outpatient clinics at the University of Illinois Medical Center and via flyers posted in the community. Participants in the ASD group met criteria for an ASD on the Autism Diagnostic Observation Schedule (ADOS^23^). The 15 of 17 ASD participants with a parent available to provide historical information also met criteria for an ASD on the Autism Diagnostic Inventory-Revised (ADI-R^24^). Participants in the ASD group received a consensus DSM-IV-TR clinical diagnosis of Autistic Disorder (*n*=7), Asperger's Disorder (*n*=9), or Pervasive Developmental Disorder-Not Otherwise Specified (PDD-NOS; *n*=1). There were no performance differences on reversal learning tasks amongst the three diagnostic groups, and therefore ASD participants were pooled for statistical analyses as planned.

Control participants were recruited from the community and had a Social Communication Questionnaire score of eight or lower (SCQ^25^), no known personal history of psychiatric or neurologic disorders, and no first- or second-degree relative with a suspected ASD or other familial neuropsychiatric illness. The ASD and control groups did not differ significantly on age, gender or Full-Scale Intelligent Quotient (IQ). All participants were free of medications known to affect cognitive abilities, including antipsychotics, psychostimulants, antidepressants and anticonvulsants. Participants were at least 7 years of age and had Full-Scale, Verbal and Performance IQs⩾70.

For individuals with an ASD diagnosis, a family member completed the Repetitive Behavior Subscales-Revised (RBS-R,^26^ a questionnaire used to assess repetitive, ritualistic and obsessive-compulsive behaviors (see Table 2 for a summary of clinical characteristics of participants in the ASD group). All participants completed informed consent or assent, and study procedures were approved by the Institutional Review Board at the University of Illinois at Chicago.

### fMRI behavioral paradigms

#### Two-choice reversal learning task

**Figure 1:**
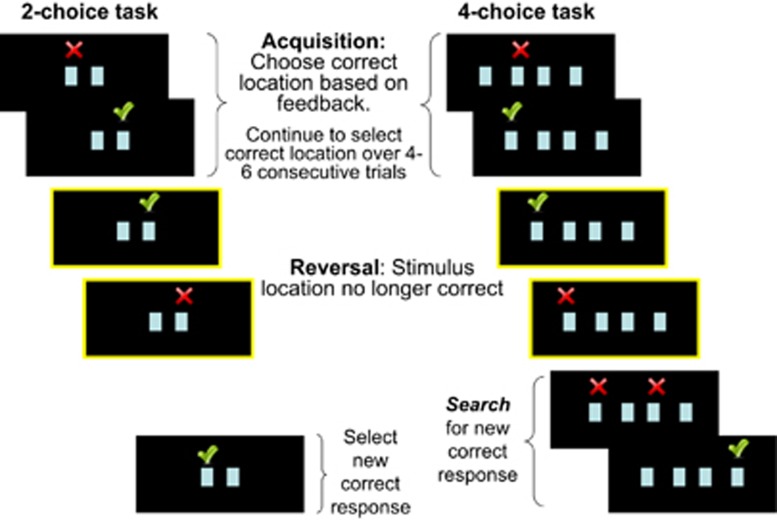
Schematic presentation of two- and four-choice reversal learning tasks. Events highlighted show trials selected to examine activation at reversal, that is, participants' response to unexpected non-reinforcement versus ongoing positive reinforcement of a learned response.

Participants were presented with two identical stimuli (one stimulus each on the left and right side of the display screen) and instructed to select the stimulus that was in the correct location by pressing a button corresponding to its location on the screen (Figure 1). Participants used both hands to hold a four-button box placed on their torso. Participants used the two outer buttons to indicate their response choice (left button for stimulus on the left, and right button for right stimulus choice). Immediate feedback was provided in the form of check marks (correct) or crosses (incorrect), which appeared directly above the stimulus selected until the end of the trial.

Requirements to change response set were imposed by making the other stimulus location the correct response choice. In order to reduce the predictability of the reversal in reinforcement contingencies, and therefore the predictability of receiving negative feedback on a given trial, the correct location changed after a variable number (four to six) of consecutive correct responses. Each trial (including presentation of stimulus, participant response, and feedback presentation) lasted for 2.5 s, followed by a 500 ms intertrial interval during which a blank screen was presented. One hundred eighty trials were presented over a fixed task duration of 9 min.

#### Four-choice reversal learning task

In the four-choice task, participants were presented with four identical stimuli placed along the horizontal axis of the display screen (Figure 1). They were told to choose the stimulus that was in the correct location, this time using all four response buttons. Two buttons were assigned to each hand. Each of the four stimulus locations had an equal probability of being the correct stimulus choice.

The four- and the two-choice tasks were similar, with the following two exceptions. First, in the four-choice task, in order to reduce demands on working memory imposed by having to keep track of which locations were previously determined to be incorrect response choices, feedback indicating that a response choice was incorrect remained on screen until participants selected the new correct location in a subsequent trial. Second, this paradigm incorporated a predetermined rate of incorrect trials at the point of reversal to ensure similar rates of non-reinforcement among participants at the reversal. The first choice of the three alternative choices was correct on 15% of trials, the second choice was correct on 33% of trials and the third and final choice was always correct. The two- and four-choice tasks were presented in counterbalanced order across participants. There was no effect of the order of task presentation on brain activity or behavioral measures of task performance, and thus task order was not considered a factor in data analysis.

### MRI image acquisition

MRI studies were performed using a 3.0 tesla whole-body scanner with a standard quadrature coil (Signa, General Electric Medical System, Milwaukee, WI, USA). Functional images were acquired using a single shot gradient-echo echo-planar imaging sequence (15 axial slices; TR=1000 ms; TE=25 ms; flip angle=90° slice thickness=5 mm; gap=1 mm; acquisition matrix=64 × 64; voxel size=3.12 mm × 3.12 mm × 5 mm; field of view (FOV)=20 × 20 cm^2^; 540 images). This protocol provided a FOV typically extending from the dorsal neocortex to dorsal pons, and therefore covered the neocortical and striatal regions of primary interest. Anatomical images collected to align and register the functional images were acquired with a three-dimensional volume inversion recovery fast spoiled gradient-recalled at steady state pulse sequence (120 axial slices; flip angle=25° slice thickness=1.5 mm; gap=0 mm; FOV=24 × 24 cm^2^).

### Image preprocessing and analysis

Event-related fMRI analyses were carried out using FSL 4.1.0 (FMRIB Software Library;^27^ with the FEAT (fMRI Expert Analysis Tool) and Randomize (http://www.fmrib.ox.ac.uk/fsl/randomize) tools. Brain Extraction Tool (BET) software was used to remove non-brain tissue from structural images.^28^ MCFLIRT motion correction was applied to functional data sets.^29^ A high-pass temporal filter with a cutoff of 100 ms was applied to the data. Spatial smoothing was conducted using a Gaussian kernel of full-width half-maximum 6 mm. Functional data were registered to the high-resolution structural scan, and then transformed into standard MNI (Montreal Neurological Institute) space using the MNI152 template.

### Modeling of activation responses

The time of onset of performance feedback, which immediately followed response choices, was used to identify the trial-wise events of interest for event-related analysis of the functional time-series data. As indicated in Figure 1, the following epochs of the time-series data were modeled in both the two- and four-choice reversal learning tasks at the onset of two types of events for the duration of feedback presentation to intertrial interval: (1) the first instance of non-reinforcement for a learned response at reversal (indicating that participants' previous response set was no longer correct); and (2) when participants received expected reinforcement following correct responses (that is, reinforcement of the second consecutive correct response and all later correct responses in a set). The difference between these two events was the primary measure of interest for each participant. A double-gamma hemodynamic response function was applied to each model. In an exploratory analysis, age was included as a covariate in all imaging analyses but results were not appreciably different from the primary analyses.

In order to examine brain activation related to processing unexpected non-reinforcement and planning a behavioral reversal, responses to unexpected non-reinforcement and expected reinforcement were contrasted separately for the two- and four-choice tasks. For group analyses, FSL's Randomize v2.1 tool was used to generate a test statistic map through permutation-based non-parametric testing which corrects for multiple comparisons.^30^ Threshold-Free Cluster Enhancement (TFCE^31^) was used to identify significant clusters of activation. Specifically, for each group, a non-parametric one-sample *t*-test with variance smoothing of 6 mm full-width half-maximum, and TFCE with an experiment-wise Type 1 error rate of *P*<.01, were used to identify clusters of statistically significant activity at reversal. To identify differences in activity at reversal between the ASD and control groups, a non-parametric two-sample *t*-test with 500 permutations, and the same TFCE procedure and parameters were applied.

### Performance measures on the reversal learning tasks

In both the two- and four-choice tasks, the total number of reversals completed overall, as well as the number of incorrect and correct responses made in each set, were recorded for each participant. In both the four- and two-choice tasks, the ASD and control groups did not differ in the mean number of reversals completed (four-choice task: ASD group (mean=23.7, s.d.=1.7), controls (mean=24.3, s.d.=1.8); two-choice task ASD group (mean=27.8, s.d.=4.6) and controls (mean=30.0, s.d.=3.7).

Errors following a reversal were classified as either perseverative errors or failures to maintain set, as in our previous study.^3^ Perseverative errors occurred after reversal in the response-outcome contingency, when participants chose the previously reinforced response before choosing the new correct response. Failures to maintain set occurred when participants chose the previously reinforced response after having selected the new correct choice at least once. Thus, the number of perseverative errors provided an index of how quickly a participant shifted their response after reversal, whereas the number of failures to maintain set provided a measure of how consistently the new correct choice pattern was maintained.

## Results

### Imaging results

#### Activation during the four-choice reversal learning task

**Figure 2:**
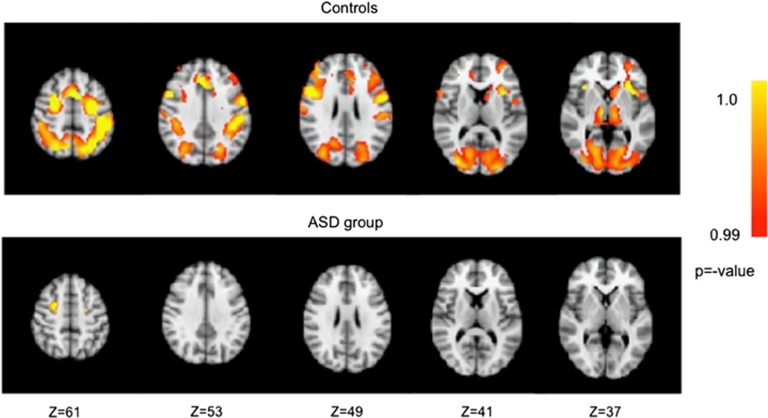
Activation in controls and ASD participants for the contrast of unexpected non-reinforcement versus expected positive reinforcement of a learned response in the four-choice task. ASD, autism spectrum disorder.

In controls, significant activation at reversal during the four-choice task was present bilaterally in ventral striatum, thalamus, insula, motor, and affective subdivisions of anterior cingulate, dorsolateral prefrontal cortex, premotor cortex, pre-supplementary motor area, posterior parietal cortex, primary visual cortex, lateral extrastriate cortex and precuneus, and in the left cognitive subdivision of anterior cingulate, left caudate, and left orbitofrontal cortex. In the ASD group, significant activation at reversal was observed in bilateral premotor cortex. See Figure 2 and Table 3 for a summary of activation for both groups for the four-choice task. Table 3 presents regions in the four-choice reversal learning task showing significant activation at reversal compared to expected positive reinforcement for ASD.

#### Activation during the two-choice reversal learning control task

For controls in the two-choice reversal learning task, in which the requirements for planning a new response were minimal as the necessary alternative response was clear, non-reinforcement of learned responses relative to expected reinforcement of correct responses at reversal trials led to significant activation in bilateral primary visual cortex only. The ASD group showed significant activation in the two-choice task at reversal in left motor cingulate cortex, left premotor cortex, and in bilateral posterior parietal cortex. Activation for both groups is summarized in Table 4 below.

#### Group comparison of activation during the reversal learning tasks

**Figure 3:**
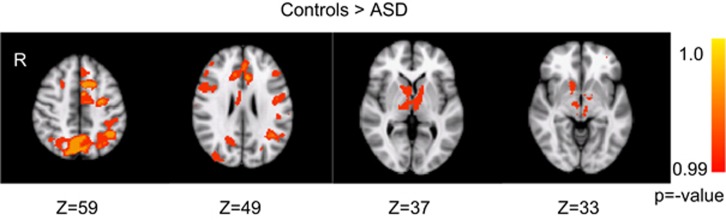
Regions for which significantly reduced activation was observed in the autism spectrum disorder (ASD) group compared with the control group, for the contrast of unexpected non-reinforcement versus expected positive reinforcement of a learned response in the four-choice task.

Group comparisons were performed to identify brain regions with differential activation in the ASD group versus controls when reversing a learned response to an alternative response with an uncertain outcome on the four-choice task. Individuals with ASD showed reduced activation relative to controls at reversal in the following regions: ventral striatum, thalamus, motor, cognitive and affective subdivisions of anterior cingulate, premotor cortex, pre-supplementary motor area, posterior parietal cortex, lateral extrastriate cortex and precuneus, and left dorsolateral prefrontal cortex (Figure 3; Table 5). There were no group differences in activation at reversal on the two-choice task.

### Behavioral performance

In the two- and four-choice tasks, the ASD and control groups did not differ in the mean number of reversals completed (F(1,38)=1.36, *P*=0.25; see Table 6 for a summary of performance measures). The ASD and control groups did not differ in their rates of perseverative errors or failures to maintain set on either the two- or four-choice task, nor were there group differences on any response latency measure.

### Clinical correlations

In exploratory analyses, for each participant in the ASD group, the peak activation during reversal in regions where significant group differences were determined was correlated with clinical ratings of behavioral and cognitive rigidity (ADI-C subscale, and RBS-R subscale and total scores). No significant relationship was found. There were no significant relationships between other clinical ratings of ASD or demographic measures with measures of brain activation.

## Discussion

The current fMRI study used a reversal learning paradigm to examine the functional integrity of brain circuitry supporting flexible choice behavior in ASD. When changing from a learned response preference to a new response choice with uncertain outcome, the ASD group demonstrated reduced activation relative to controls in brain regions supporting (1) cognitive decision-making processes, including frontal motor planning systems, parietal cortex, and the cognitive subdivision of anterior cingulate cortex, and (2) reinforcement learning processes, including ventral striatum and the affective subdivision of anterior cingulate cortex.

Deficits in the interaction between these two systems may contribute to difficulty shifting from a learned to a newly adaptive response. For instance, an attenuated response in ventral striatum to non-reinforcement cues that signal a need to change behavior may contribute to reduced bottom-up drive to rostral frontal and dorsal parietal attention and alerting systems, and a subsequent failure to attend to possible new response options. A reduced response to non-reinforcement could also impair cognitive and motor planning processes, and result in a failure to disengage from a preferred response in order to initiate new adaptive behaviors. To our knowledge, this study is the first to provide clarification about impaired functioning in the brain systems underlying flexible choice behavior in ASD, and enhances understanding the neurocognitive substrates of behavioral rigidity, a clinically relevant target for treatment.

Importantly, the functional deficits we observed in the ASD group in frontostriatal and parietal systems were specific to task conditions in which the outcomes of future choice behaviors were uncertain; deficits were not seen when the outcomes of new response patterns were fully predictable. The present findings are comparable to a recent study showing under activation of medial prefrontal cortex in ASD during reversal learning that involved probabilistic reinforcement.^12^ The current study extends these recent findings by showing that changes in brain systems in ASD are not simply altered when a shift in choice patterns is required, but only occur when a learned choice pattern must be inhibited and new choice options have an uncertain outcome. Thus, from a clinical perspective, behavioral inflexibility may be particularly pronounced when individuals must stop an ongoing behavioral pattern and choose new options from several alternatives. This may contribute to anxiety^32^ and a worsening of rigid behavior and an increased need for sameness in novel situations in which the outcomes of future behaviors are ambiguous.

We did not detect altered activation in one single distinct brain circuit in ASD in relation to implementing new choice behaviors after reversal. Instead, our findings indicate a deficit in both cognitive and motivational salience systems, both of which are necessary in order to successfully and flexibly adapt behavior to changing environmental contingencies. Parsing out the role of the components of these circuits in flexible choice behavior may shed further light on the causes of behavioral rigidity in ASD.

### Cognitive and motor planning during reversal learning

Successful reversal learning requires several interacting cognitive processes including the ability to inhibit learned, prepotent response tendencies and to select and engage in new adaptive behaviors. When participants reversed learned responses in the condition when performance outcomes were uncertain, we observed activation in typically developing control participants in regions known to be involved in motor planning and attention, including dorsolateral prefrontal, premotor and posterior parietal cortices. Significantly reduced activation of these regions in the ASD group indicates a deficit in recruiting the neurocognitive systems necessary for withholding learned response patterns and planning and enacting new adaptive responses.

Reduced activation of dorsolateral prefrontal cortex in the ASD group may indicate impairments in a number of cognitive processes supported by this region that are necessary for flexibly updating behavior. The role of dorsolateral prefrontal cortex in withholding prepotent response tendencies^33, 34, 35^ allows for the selection and implementation of new response choices. Thus, a deficit in the inhibition of previously learned responses may contribute to difficulty disengaging from preferred responses in ASD. This is consistent with previous reports of response inhibition deficits in ASD^36, 37, 38^ and their relation to restricted and repetitive behaviors in ASD.^4^ To our knowledge, the findings in the present study are the first to indicate a neural mechanism by which such deficits might adversely impact the ability to engage in new choice behaviors.

A similarly reduced activation in ASD at reversal was seen in posterior parietal cortex and precuneus, which have a prominent role in supporting visual attention processes.^39, 40^ In ASD, a lack of adequate attention to alternative response options may reduce the likelihood that individuals attend to and select alternative response patterns over known and preferred alternatives. This could sustain learned response preferences over available alternative responses, and contribute to rigid patterns of behavior in ASD.

The ASD group also showed reduced activation at reversal in a number of regions involved in motor planning. For instance, reduced activity was seen in the pre-supplementary motor area, which is important in generating and updating motor plans.^41, 42^ Involvement of this region is also observed in tasks where there is a concomitant shift in both response strategy and behavior, and it is believed to integrate cognitive decision-making processes with corresponding shifts in response plans.^43, 44^ Our findings suggest that alterations in the function of the pre-supplementary motor area may result in difficulty in deciding to shift behavior and subsequently in planning new responses, which may contribute to reduced behavioral flexibility in ASD.

The ASD group also showed reduced activation in dorsal motor and cognitive subdivisions of the cingulate cortex.^45^ Like the pre-supplementary motor area, motor cingulate cortex supports the integration of cognitive decision-making processes with motor planning, and has been shown to be engaged during reversal of conditioned associations.^46^ Activation of the cognitive subdivision of anterior cingulate cortex is consistently reported in tasks requiring action selection and decision-making in the context of competing attentional demands (for a review, see Bush *et al.*^47^). Functional alterations in the cognitive division of anterior cingulate cortex in ASD are commonly reported in tasks involving attention to error and performance monitoring.^48, 49^ Clinically, diminished attention to novel or unexpected events could sustain well-established patterns of behavior over newly adaptive responses. Taken together, reduced activation in dorsal cingulate regions at reversal may suggest a deficit in managing competing information regarding possible alternative response choices, and disrupt the ability to effectively engage in new behavioral plans.

### Reinforcement learning during reversal

The ability to appropriately respond to unexpected non-reinforcement, which cues participants to change a learned behavior to a new adaptive response, is crucial for reversal learning. In the present study, during reversal trials on the four-choice task, that is, when expected reinforcement was not received, reduced activation in the ASD group relative to controls was observed in the ventral striatum and the affective subdivision of the anterior cingulate. Our results suggest that there may be a reduced alerting response to behavioral consequences that motivate a need for change in behavior, suggesting a reduced sensitivity to reinforcement cues in ASD. This is consistent with recent studies that suggest a reduced response in ventral striatum and anterior cingulate to secondary reinforcers such as positive social cues, money and personally rewarding stimuli in individuals with ASD.^50, 51, 52, 53, 54^ The current results extend these previous findings to suggest that non-reinforcement also triggers a less robust response in ventral striatal and affective cingulate reward circuits in ASD. Altogether, these results suggest deficits in responding to, and learning from, a broad range of reinforcers in ASD. As such, reward-processing deficits may have a specific role in rigid behavior in ASD by sustaining learned responses even in the face of feedback that conveys that an alternative response strategy would be more likely to be positively reinforced.

### Integration of cognitive and affective processes in behavioral flexibility

The pattern of functional alterations in cognitive decision-making and motor response planning regions, together with deficits in the limbic circuitry supporting reinforcement learning, raises the possibility that an impaired interaction of these systems may contribute to behavioral flexibility deficits in ASD. The ventral striatal and affective cingulate deficits in the ASD group may indicate reduced bottom-up drive from limbic circuitry in response to unexpected non-reinforcement that typically provides an important motivational drive to dorsal cognitive systems to change behavior.

A direct link between reward processing and the motor planning, and attentional components of behavioral control has been reported in both humans and non-human primates.^55, 56^ In addition, several studies have shown that as the relevance of external cues for directing future behavior increases, there is an associated increase in activation in dorsal premotor and parietal attention systems.^20, 57, 58^ Repetitive patterns of behavior may occur in individuals with ASD if information regarding response-outcome contingencies fails to provide sufficient motivational salience to initiate planning for potential new adaptive actions.

### The role of outcome uncertainty on behavioral flexibility in autism spectrum disorder

Our study was designed to examine flexible choice behavior in ASD under circumstances in which the outcomes of response choices were certain and uncertain. In a previous study using the same tasks in typically developing control participants,^20^ we demonstrated that greater uncertainty of future outcomes resulted in increased activation in the dorsal and ventral frontostriatal circuitry. In the present study, during behavioral reversals in the four-choice task only, that is, reversals during circumstances when response outcomes were uncertain, did the ASD group demonstrate a reduced response in frontal, striatal, and parietal systems. This suggests that uncertainty of future outcomes may significantly affect flexible choice behavior in ASD. This could contribute to a worsening of rigid behavior in novel or unexpected situations in ASD when adapting behavior to uncertain circumstances.

### Conclusions

In summary, to our knowledge, this is the first study to identify specific functional alterations in brain circuitry during response shifting in ASD, and to suggest mechanisms by which these deficits may contribute to clinical manifestations of behavioral rigidity and ‘insistence on sameness'. The results suggest that in ASD, functional impairments in limbic, attentional, and response planning brain systems contribute to behavioral rigidity when environmental cues signal a need to change behavior. Reduced drive from ventral striatum could fail to provide sufficient bottom-up drive to attention and motor planning areas, and thus impede the flexible selection and planning of future behavior. This could manifest clinically as rigid patterns of behavior that are not contextually adaptive and are characteristic of ASD. Studies of reversal learning can be readily conducted in rodent models, and thus may represent a useful translational strategy for testing mechanistic hypotheses about the neurobiology of behavioral rigidity and its pharmacologic treatment.^59^ As such, our findings inform understanding of a clinical dimension of ASD for which effective treatments are not yet available, and provide a promising neurobehavioral strategy that may be especially useful in translational research programs.

## Figures and Tables

**Table 1:** Demographic and cognitive characteristics of study participants

	*ASD group (*n*=17, 5 females)*	*Controls (*n*=23, 5 females)*	*Significance*
Age (years)	17.4 (8.6), 9–44	18.6 (8.4), 7–38	NS
Full-scale IQ	103.9 (15.5), 87–140	110.9 (9.9), 95–133	NS
Verbal IQ	100.4 (15.9), 71–120	113.0 (10.6), 93–133	*P*=0.004
Performance IQ	106.7 (16.6), 84–145	107.5 (9.3), 91–128	NS

Abbreviations: ASD, autism spectrum disorder; IQ, intelligent quotient; NS, not significant.

**Table 2:** Clinical characteristics of ASD study participants

*Clinical measure*	*ASD group scores*
*Autism Diagnostic Interview—revised*	
A—Social interaction	20.0 (5.8), 9–29
B—Communication and language	14.7 (4.2), 10–25
C—Restricted and repetitive behaviors	6.3 (2.4), 3–11
D—Severity	2.5 (1.4), 0–5
	
*Repetitive Behavior Scale—revised*	
Stereotypies	3.2 (2.9), 0–8
Self-injury	2.4 (2.6), 0–8
Compulsions	3.1 (3.6), 0–11
Rituals	4.8 (4.7), 0–18
Sameness	9.9 (8.0), 1–28
Restricted interests	4.2 (2.7), 0–10
Total score	27.7 (20.1), 5–69

Abbreviation: ASD, autism spectrum disorder.

**Table 3:** Summary of regions in the four-choice reversal learning task showing significant activation at reversal compared to expected positive reinforcement for ASD and controls

*Region*	*Hemisphere*	*Controls*				*ASD*			
		*Max.* t*-value*	*Co-ordinates*			*Max.* t*-value*	*Co-ordinates*		
			x	y	z		x	y	z
Ventral striatum	R	5.14	16	14	−6	—	—	—	—
	L	3.84	−14	14	−6	—	—	—	—
Thalamus	R	5.60	10	−18	4	—	—	—	—
	L	5.18	−10	−20	4	—	—	—	—
Dorsal caudate	L	3.76	−10	8	6	—	—	—	—
Orbitofrontal cortex	L	3.88	−28	58	−12	—	—	—	—
Insula	R	6.70	34	26	−6	—	—	—	—
	L	7.86	−30	22	−6	—	—	—	—
Anterior cingulate cortex, motor division	R	8.81	2	16	40	—	—	—	—
	L	8.87	−2	16	42	—	—	—	—
Anterior cingulate cortex, cognitive division	R	—	—	—	—	—	—	—	—
	L	3.76	−8	22	24	—	—	—	—
Anterior cingulate cortex, affective division	R	4.92	10	38	20	—	—	—	—
	L	3.66	−8	32	20	—	—	—	—
Dorsolateral prefrontal cortex	R	4.04	40	26	26	—	—	—	—
	L	5.47	−46	30	30	—	—	—	—
Premotor cortex	R	7.41	52	8	26	—	—	—	—
	L	7.38	−56	2	30	—	—	—	—
Pre-supplementary motor area	R	4.19	8	0	56	6.37	26	0	50
	L	5.56	−10	2	54	5.45	−24	−6	50
Posterior parietal cortex	R	7.07	46	−30	42	—	—	—	—
	L	8.93	−46	−36	46	—	—	—	—
Primary visual cortex	R	5.15	18	−74	8	—	—	—	—
	L	5.22	−10	−90	0	—	—	—	—
Lateral extrastriate cortex	R	6.38	28	−66	44	—	—	—	—
	L	6.61	−16	68	54	—	—	—	—
Precuneus	R	6.61	10	−70	46	—	—	—	—
	L	5.72	−10	−68	52	—	—	—	—

Abbreviations: ASD, autism spectrum disorder; L, left; R, right.

**Table 4:** Regions in the two-choice reversal learning task showing significant activation at reversal compared with expected positive reinforcement for ASD and control participants

*Region*	*Hemisphere*		*Controls*				*ASD*		
		*Max.* t*-value*	*Co-ordinates (MNI)*			*Max.* t*-value*	*Co-ordinates (MNI)*		
			x	y	z		x	y	z
Motor cingulate	L	—	—	—	—	4.60	−6	6	44
Premotor cortex	L	—	—	—	—	5.47	−58	6	28
Posterior parietal cortex	R	—	—	—	—	4.49	44	−30	44
	L	—	—	—	—	5.21	−40	−32	44
Primary visual cortex	R	5.63	12	−76	−6	—	—	—	—
	L	4.91	−14	−70	−6	—	—	—	—

Abbreviations: ASD, autism spectrum disorder; L, left; MNI, Montreal Neurological Institute; R, right.

**Table 5:** Regions for which activation in the four-choice task at reversal was greater in the control group than in the ASD group

*Region*	*Hemisphere*	*Max.* t*-value*	*Co-ordinates (MNI space)*		
			x	y	z
Ventral striatum	R	2.98	10	8	−2
	L	—	—	—	—
Thalamus	R	3.05	12	−18	6
	L	3.15	−6	−8	4
Anterior cingulate cortex, motor division	R	3.40	2	16	40
	L	4.05	−10	12	40
Anterior cingulate cortex, cognitive division	R	3.43	2	34	30
	L	3.59	−2	36	30
Anterior cingulate cortex, affective division	R	3.56	2	36	20
	L	3.86	−2	40	20
Dorsolateral prefrontal cortex	R	—	—	—	—
	L	5.06	−46	30	30
Premotor cortex	R	3.31	40	−8	34
	L	4.29	−28	10	42
Pre-supplementary motor area	R	3.59	2	20	56
	L	3.45	−2	20	56
Posterior parietal cortex	R	2.96	42	−38	46
	L	4.10	−42	−54	46
Lateral extrastriate cortex	R	4.52	32	86	14
	L	—	—	—	—
Precuneus	R	4.40	4	−74	46
	L	3.46	−4	−74	46

Abbreviations: ASD, autism spectrum disorder; L, left; MNI, Montreal Neurological Institute; R, right.

**Table 6:** Summary of performance measures for ASD participants and controls on the four- and two-choice reversal learning tasks

	*ASD Group*	*Controls*	*F(1,38)*	P*-value*
*Four-choice task*				
Number of reversals	23.7 (1.7), 20–26	24.3 (1.8), 19–26	1.36	0.25
Perseverative errors	1.1 (1.7), 0–6	0.5 (1.3), 0–5	1.58	0.22
Failures to maintain set	4.7 (5.3), 0–17	3.0 (5.1), 0–22	0.99	0.33
				
*Two-choice task*				
Number of reversals	27.8 (4.6), 13–33	30.0 (3.7), 21–33	2.45	0.12
Perseverative errors	7.1 (9.0), 0–38	3.2 (4.6), 0–17	3.27	0.08
Failures to maintain set	7.8 (9.0), 0–40	4.4 (6.1), 0–24	1.98	0.17

Abbreviations: ASD, autism spectrum disorder.
